# Supplementary material for: Unveiling the Bioactive Potential of the Invasive Jellyfish Phyllorhiza punctata Through Integrative Transcriptomic and Proteomic Analyses
Source: Biomolecules. 2025 Aug 4;15(8):1121. doi: 10.3390/biom15081121 (PMC12383608; doi:10.3390/biom15081121)
Supplement: Supplementary file 1 [file biomolecules-15-01121-s001.zip › Suplementary Material/FileS3/PEPFOLD4/XP_065051314_5193/clusters.html]

 PEP-FOLD clusters


Download archive of all models  here   

|  |  |  |
| --- | --- | --- |
| # | file | sOPEP |
| # cluster 1 - Size: 5 |  |  |
|  | XP\_065051314\_5193-model1 | -35.8016 |
|  | XP\_065051314\_5193-model1.1 | -35.7165 |
|  | XP\_065051314\_5193-model1.2 | -35.5058 |
|  | XP\_065051314\_5193-model1.3 | -35.3437 |
|  | XP\_065051314\_5193-model1.4 | -33.9608 |
| # cluster 2 - Size: 1 |  |  |
|  | XP\_065051314\_5193-model2 | -34.9928 |
| # cluster 3 - Size: 5 |  |  |
|  | XP\_065051314\_5193-model3 | -33.4361 |
|  | XP\_065051314\_5193-model3.1 | -32.326 |
|  | XP\_065051314\_5193-model3.2 | -32.2481 |
|  | XP\_065051314\_5193-model3.3 | -32.1888 |
|  | XP\_065051314\_5193-model3.4 | -32.0388 |
| # cluster 4 - Size: 1 |  |  |
|  | XP\_065051314\_5193-model4 | -33.2803 |
| # cluster 5 - Size: 1 |  |  |
|  | XP\_065051314\_5193-model5 | -33.0612 |
| # cluster 6 - Size: 34 |  |  |
|  | XP\_065051314\_5193-model6 | -33.0309 |
|  | XP\_065051314\_5193-model6.1 | -32.8999 |
|  | XP\_065051314\_5193-model6.2 | -32.7539 |
|  | XP\_065051314\_5193-model6.3 | -32.5636 |
|  | XP\_065051314\_5193-model6.4 | -32.5348 |
|  | XP\_065051314\_5193-model6.5 | -32.5335 |
|  | XP\_065051314\_5193-model6.6 | -32.4669 |
|  | XP\_065051314\_5193-model6.7 | -32.4248 |
|  | XP\_065051314\_5193-model6.8 | -32.3477 |
|  | XP\_065051314\_5193-model6.9 | -32.3126 |
|  | XP\_065051314\_5193-model6.10 | -32.1974 |
|  | XP\_065051314\_5193-model6.11 | -32.1304 |
|  | XP\_065051314\_5193-model6.12 | -32.1276 |
|  | XP\_065051314\_5193-model6.13 | -32.1241 |
|  | XP\_065051314\_5193-model6.14 | -32.1159 |
|  | XP\_065051314\_5193-model6.15 | -32.0885 |
|  | XP\_065051314\_5193-model6.16 | -31.9967 |
|  | XP\_065051314\_5193-model6.17 | -31.9195 |
|  | XP\_065051314\_5193-model6.18 | -31.8828 |
|  | XP\_065051314\_5193-model6.19 | -31.8765 |
|  | XP\_065051314\_5193-model6.20 | -31.7897 |
|  | XP\_065051314\_5193-model6.21 | -31.7802 |
|  | XP\_065051314\_5193-model6.22 | -31.7781 |
|  | XP\_065051314\_5193-model6.23 | -31.706 |
|  | XP\_065051314\_5193-model6.24 | -31.6997 |
|  | XP\_065051314\_5193-model6.25 | -31.5772 |
|  | XP\_065051314\_5193-model6.26 | -31.5694 |
|  | XP\_065051314\_5193-model6.27 | -31.5231 |
|  | XP\_065051314\_5193-model6.28 | -31.3992 |
|  | XP\_065051314\_5193-model6.29 | -31.327 |
|  | XP\_065051314\_5193-model6.30 | -31.3138 |
|  | XP\_065051314\_5193-model6.31 | -31.2268 |
|  | XP\_065051314\_5193-model6.32 | -30.8871 |
|  | XP\_065051314\_5193-model6.33 | -30.8459 |
| # cluster 7 - Size: 1 |  |  |
|  | XP\_065051314\_5193-model7 | -32.7286 |
| # cluster 8 - Size: 9 |  |  |
|  | XP\_065051314\_5193-model8 | -32.7144 |
|  | XP\_065051314\_5193-model8.1 | -32.6351 |
|  | XP\_065051314\_5193-model8.2 | -32.5343 |
|  | XP\_065051314\_5193-model8.3 | -32.425 |
|  | XP\_065051314\_5193-model8.4 | -32.0642 |
|  | XP\_065051314\_5193-model8.5 | -32.0631 |
|  | XP\_065051314\_5193-model8.6 | -31.9759 |
|  | XP\_065051314\_5193-model8.7 | -31.9645 |
|  | XP\_065051314\_5193-model8.8 | -31.4917 |
| # cluster 9 - Size: 1 |  |  |
|  | XP\_065051314\_5193-model9 | -32.5762 |
| # cluster 10 - Size: 2 |  |  |
|  | XP\_065051314\_5193-model10 | -32.5587 |
|  | XP\_065051314\_5193-model10.1 | -31.2715 |
| # cluster 11 - Size: 3 |  |  |
|  | XP\_065051314\_5193-model11 | -32.1094 |
|  | XP\_065051314\_5193-model11.1 | -31.65 |
|  | XP\_065051314\_5193-model11.2 | -31.6116 |
| # cluster 12 - Size: 7 |  |  |
|  | XP\_065051314\_5193-model12 | -32.1052 |
|  | XP\_065051314\_5193-model12.1 | -31.9209 |
|  | XP\_065051314\_5193-model12.2 | -31.8993 |
|  | XP\_065051314\_5193-model12.3 | -31.7445 |
|  | XP\_065051314\_5193-model12.4 | -31.6898 |
|  | XP\_065051314\_5193-model12.5 | -31.628 |
|  | XP\_065051314\_5193-model12.6 | -30.4411 |
| # cluster 13 - Size: 3 |  |  |
|  | XP\_065051314\_5193-model13 | -32.1032 |
|  | XP\_065051314\_5193-model13.1 | -32.0248 |
|  | XP\_065051314\_5193-model13.2 | -30.5503 |
| # cluster 14 - Size: 2 |  |  |
|  | XP\_065051314\_5193-model14 | -31.9748 |
|  | XP\_065051314\_5193-model14.1 | -31.2108 |
| # cluster 15 - Size: 5 |  |  |
|  | XP\_065051314\_5193-model15 | -31.6338 |
|  | XP\_065051314\_5193-model15.1 | -31.1558 |
|  | XP\_065051314\_5193-model15.2 | -30.7576 |
|  | XP\_065051314\_5193-model15.3 | -30.7508 |
|  | XP\_065051314\_5193-model15.4 | -29.7892 |
| # cluster 16 - Size: 6 |  |  |
|  | XP\_065051314\_5193-model16 | -31.5456 |
|  | XP\_065051314\_5193-model16.1 | -31.3946 |
|  | XP\_065051314\_5193-model16.2 | -31.3154 |
|  | XP\_065051314\_5193-model16.3 | -31.03 |
|  | XP\_065051314\_5193-model16.4 | -30.9091 |
|  | XP\_065051314\_5193-model16.5 | -30.6487 |
| # cluster 17 - Size: 4 |  |  |
|  | XP\_065051314\_5193-model17 | -31.3435 |
|  | XP\_065051314\_5193-model17.1 | -30.431 |
|  | XP\_065051314\_5193-model17.2 | -30.3057 |
|  | XP\_065051314\_5193-model17.3 | -29.8788 |
| # cluster 18 - Size: 1 |  |  |
|  | XP\_065051314\_5193-model18 | -31.2264 |
| # cluster 19 - Size: 1 |  |  |
|  | XP\_065051314\_5193-model19 | -31.1174 |
| # cluster 20 - Size: 2 |  |  |
|  | XP\_065051314\_5193-model20 | -31.0157 |
|  | XP\_065051314\_5193-model20.1 | -30.5665 |
| # cluster 21 - Size: 1 |  |  |
|  | XP\_065051314\_5193-model21 | -30.8892 |
| # cluster 22 - Size: 3 |  |  |
|  | XP\_065051314\_5193-model22 | -29.4875 |
|  | XP\_065051314\_5193-model22.1 | -29.3722 |
|  | XP\_065051314\_5193-model22.2 | -28.4302 |
| # cluster 23 - Size: 2 |  |  |
|  | XP\_065051314\_5193-model23 | -28.7259 |
|  | XP\_065051314\_5193-model23.1 | -27.9625 |
|  |  |  |
